# Bayesian modeling of item heterogeneity in dichotomous recognition memory data and prospects for computerized adaptive testing

**DOI:** 10.1038/s41598-022-04997-3

**Published:** 2022-01-24

**Authors:** Jeremie Güsten, David Berron, Emrah Düzel, Gabriel Ziegler

**Affiliations:** 1 German Center for Neurodegenerative Diseases, Magdeburg, Germany; 2 Institute of Cognitive Neurology and Dementia Research, Otto-von-Guericke University, Magdeburg, Germany; 3 Clinical Memory Research Unit, Department of Clinical Sciences Malmö, Lund University, Lund, Sweden; 4 Institute of Cognitive Neuroscience, University College London, London, UK

**Keywords:** Psychology, Cognitive neuroscience, Diagnosis

## Abstract

Most current models of recognition memory fail to separately model item and person heterogeneity which makes it difficult to assess ability at the latent construct level and prevents the administration of adaptive tests. Here we propose to employ a General Condorcet Model for Recognition (GCMR) in order to estimate ability, response bias and item difficulty in dichotomous recognition memory tasks. Using a Bayesian modeling framework and MCMC inference, we perform 3 separate validation studies comparing GCMR to the Rasch model from IRT and the 2-High-Threshold (2HT) recognition model. First, two simulations demonstrate that recovery of GCMR ability estimates with varying sparsity and test difficulty is more robust and that estimates improve from the two other models under common test scenarios. Then, using a real dataset, face validity is confirmed by replicating previous findings of general and domain-specific age effects (Güsten et al. in Cortex 137:138–148, 10.1016/j.cortex.2020.12.017, 2021). Using cross-validation we show better out-of-sample prediction for the GCMR as compared to Rasch and 2HT model. In addition, we present a hierarchical extension of the model that is able to estimate age- and domain-specific effects directly, without recurring to a two-stage procedure. Finally, an adaptive test using the GCMR is simulated, showing that the test length necessary to obtain reliable ability estimates can be significantly reduced compared to a non-adaptive procedure. The GCMR allows to model trial-by-trial performance and to increase the efficiency and reliability of recognition memory assessments.

## Introduction

Research on human memory has long seen a quest for adequate cognitive models to describe the relevant processes underlying behavioural responses. Modeling of recognition memory has largely seen two classes of stochastic models: signal-detection theory (SDT) models^2^ and threshold models^3^. Both make predictions about the latent processes leading to mnemonic decision making. SDT posits distributions over the underlying memory strength which partly overlap. Threshold models, on the other hand, assume that latent memory representations are categorical and therefore disjoint. A stimulus is either remembered or not, depending on a subject-specific threshold. When the threshold is not reached, a subject is not able to recognize or detect a stimulus, but is still able to answer correctly as a result of pure guessing. It should be noted that recent decades have seen constant developments of these recognition modeling frameworks, either extending the standard SDT model (e.g. the Unequal Variance Signal Detection model [UVSD]^4^), or combining aspects of SDT and threshold models (e.g. the Dual Process Signal Detection model [DPSD]^5^), reflecting differing assumptions about the underlying mnemonic processes.

One classic threshold model that is still being used in many recognition memory paradigms is the 2 High-Threshold (2HT) Model. It belongs to a broader class of so-called multinomial processing tree (MPT) models, whose latent processes can be visualized as paths in a decision tree. In recognition memory, where performance is typically estimated based on a single false alarm rate and a hit rate, a common threshold for recognition and rejection is often assumed in order to be identifiable^3^. The corresponding threshold parameter is then called *Pr*, and given that it can be estimated by subtracting the false alarm rate from the hit rate, it is often simply called “corrected hit rate”. The 2HT model also contains a guessing parameter called *Br*.

Importantly, it has been shown that sensitivity and response bias are theoretically independent, and thus can be separately manipulated in behavioural experiments^3^. Response biases might differ across populations and may be of relevance in clinical diagnostics of various neurological disorders. For instance, a more liberal response bias has been observed in old age^6^. Moreover, Snodgrass and Corwin^3^ found that while both amnesia and dementia (Huntington’s [HD], Parkinson’s Dementia [PD] and Alzheimer’s Dementia [AD]) patients show weaker memory compared to cognitively normal subjects, only dementia patients exhibit a more liberal response bias. AD patients’ response bias was found to be similar across different recognition tasks, while in healthy older adults it varied with task and stimulus type^7^. When comparing the 2HT model with the standard SDT model (*d*$'$), Snodgrass and Corwin^3^ noted that ability and bias parameters from the 2HT model were generally more sensitive to disease status.

Item response theory (IRT) is a psychometric framework which provides sophisticated tools to define, estimate and analyze individual differences in terms of latent (or noise-free) characteristics of participants. A typical example of such a latent psychological variable (or construct) is the ability of a participant to discriminate between items in a recognition memory task. Other examples are verbal and quantitative reasoning, which are assessed in the Graduate Record Examination (GRE) and represent an admission criterion to many graduate schools in the US and Canada. As a measurement framework, IRT provides several advantages compared to the classical test theory (CTT), upon which most conventional cognitive modeling relies^8^. Given independence of item and person-specific parameters, IRT estimates of latent constructs do not depend on a given test or item. Using IRT, measurement error can be assessed at the latent construct level, which is typically not constant across the latent dimension^9^. For instance, a test that mostly employs items of average difficulty will be more precise for subjects of average ability, as compared to very high or low ability. Indeed, the fact that measurement error depends on the participant’s level of ability, has been observed for measures of cognition^10,11^, personality^12,13^, and psychiatric symptoms^14^. IRT provides tools to model this error directly and may therefore improve clinical assessments.

One promising use of IRT in modern clinical assessment is its implementation in Computerized Adaptive Testing (CAT)^15,16^. In CAT, test items are adaptively administered to participants in an optimal fashion so as to maximize the information gain and thereby the reliability of assessments. As a consequence, using CAT can lead to much shorter test lengths and higher efficiency. Moreover, as the standard error of the estimate can be obtained on a trial-by-trial basis, it can flexibly be used as a criterion set by the experimenter in order to reach a desired level of certainty. These are some of the beneficial properties that have led to widespread use of CAT in educational (e.g. TOEFL, GRE) as well as cognitive assessment. One prominent example is the NIH Toolbox^17^, which contains two adaptive language tests: the NIH-TB Oral Reading Recognition Test and the NIH-TB Picture Vocabulary Test^18^. Finally, CAT has also gained increasing popularity in clinical assessment. For instance, it has been used to assess depression^19^, anxiety^20^ as well as personality disorders^21^. Other studies in the domain have shown its usefulness via simulated tests^22,23^. Nonetheless, despite these promising examples and benefits that CAT provides, its overall use in clinical assessment is still relatively scarce^9^.

By accounting for item and person heterogeneity and providing more sophisticated tools to account for measurement error, IRT could benefit the study of recognition memory. However, IRT was not conceived to provide psychologically plausible explanations of decision-making processes in old-new recognition tasks as is the case in the cognitive models introduced above^24,25^. For instance, IRT models do not traditionally model response bias or guessing as a person parameter, as do the 2HT or SDT models. In recent years, several authors have argued for an approach that combines mathematical cognitive models with psychometric IRT models^8,26,27^ and first combined approaches with SDT have been proposed regarding recognition memory models in particular^24,25^. Others have introduced models that merge IRT with MPT, where transition probabilities in the MPT models are the result of an IRT model process^27–29^. For instance, De Boeck and Partchev^28^ presented the IRTTree framework, that combines MPT with IRT models represented as Generalized Linear Mixed Effects Models. Other authors have proposed to describe MPT parameters by the Rasch model from IRT^26,27^. Specifically, they applied the Rasch model to an extension of the 2HT model, the General Condorcet Model (GCM). Despite the two models’ formal similarity, the 2HT is mainly used to separate latent ability from response bias, while the main purpose of the GCM is to study cultural consensus regarding the correctness of responses^30^. Hence, while in the 2HT model—as is typical in recognition memory—the expected answer is fixed by design, it is the parameter of interest in the GCM.

Here we propose a new model for old-new recognition memory further denoted as GCMR (General Condorcet Model for Recognition Memory). The GCMR combines the Rasch model from IRT with the 2HT model. As mentioned above, the 2HT is a widely used model in recognition memory, which has been suggested to possess higher sensitivity in clinical assessment when compared to *d*$'$^3^. In what follows we introduce the GCMR more formally and subsequently conduct three studies to validate the approach. In a simulation study, we explore potential advantages of the approach in two typical recognition memory task scenarios, testing under which conditions ability estimates for GCMR diverge from its constituting models, the Rasch and the 2HT model, individually. We further validate the model using a real dataset consisting of large recognition memory sample collected online via Amazon Mechanical Turk. Finally, we investigate the GCMR’s potential for CAT, such as benefits for test efficiency, time savings and valuable estimates of conditional measurement error.

## Results

### Simulation study

#### Varying test difficulty and response bias

**Figure 1:**
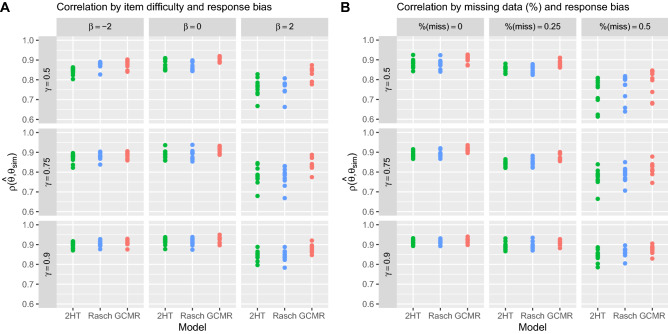
Correlation of estimated and true scores as a function of response bias and item difficulty (**A**) or missing responses (**B**). (**A**) Ground truth correlation for all models is highest when difficulty matches average ability, while it decreases for Rasch and 2HT estimates under high difficulty, especially when response bias is low. In addition, 2HT correlation with ground truth decreases under low difficulty and low bias. (**B**) While all ability estimates correlate strongly with ground truth when responses are complete, GCMR estimates show good recovery under high degree of missing responses, while ground truth correlation for Rasch and 2HT estimates decreases more strongly.

Over all 9 combinations of test difficulty and response bias, the highest mean correlation with ground truth and lowest standard error (SE) of ability estimates was observed for the GCMR ($\rho =0.885$, $\hbox {SE}=0.0065$), followed by Rasch ($\rho =0.857$, $\hbox {SE}=0.0083$) and 2HT ($\rho =0.853$, $\hbox {SE}=0.0086$) estimates, as summarized in Fig. 1A. All models exhibited high correlations with ground truth when test difficulty matched the average participant ability (GCMR: $\rho =0.912$, $\hbox {SE}=0.0048$; Rasch: $\rho =0.891$, $\hbox {SE}=0.0069$; Pr: $\rho =0.893$, $\hbox {SE}=0.0070$) and a low correlation when test difficulty was higher than average ability. The GCMR ($\rho =0.852$, $\hbox {SE}=0.0088$) showed better recovery than the Rasch ($\rho =0.794$, $\hbox {SE}=0.0126$), and 2HT ($\rho =0.796$, $\hbox {SE}=0.0125$) model. Regarding effects of response bias, all three explored models performed worst when bias was balanced (${\overline{\gamma }}=0.5$, GCMR: $\rho =0.874$, $\hbox {SE}=0.0070$, Rasch: $\rho =0.836$, $\hbox {SE}=0.0086$, 2HT: $\rho =0.827$, $\hbox {SE}=0.0095$) and best when bias was more pronounced (${\overline{\gamma }}=0.9$, GCMR: $\rho =0.904$, $\hbox {SE}=0.0053$, Rasch: $\rho =0.886$, $\hbox {SE}=0.0065$, 2HT: $\rho =0.886$, $\hbox {SE}=0.0062$).

When test difficulty was low (${\overline{\beta }} =-2$), the 2HT model was found to be more affected by lower average response bias (0.9 to 0.5) than the GCMR or the Rasch model (GCMR: $\Delta \rho =-0.026$, Rasch: $\Delta \rho = -0.029$, 2HT: $\Delta \rho = -0.053$). Under high test difficulty ($\beta =2$), both Rasch and the 2HT model were more negatively affected than GCMR (GCMR: $\Delta \rho =-0.041$, Rasch: $\Delta \rho = -0.080$, 2HT: $\Delta \rho = -0.083$).

#### Varying missingness and response bias

The proposed GCMR ($\rho =0.876$, $\hbox {SE}=0.0074$) showed a higher mean correlation and lower SE than the Rasch ($\rho =0.854$, $\hbox {SE}=0.0083$) and 2HT model ($\rho =0.885$, $\hbox {SE}=0.0065$). Again, the GCMR performed best across all explored parameter combinations in recovering its ground truth (Fig. 1B). All models showed higher correlations when missingness was low (0% missing, GCMR: $\rho =0.913$, $\hbox {SE}=0.0044$, Rasch: $\rho =0.894$, $\hbox {SE}=0.0055$, 2HT: $\rho =0.895$, $\hbox {SE}=0.0054$), and performed worst when missingness was high (50%, GCMR: $\rho =0.826$, $\hbox {SE}=0.0128$, Rasch: $\rho =0.802$, $\hbox {SE}=0.0135$, 2HT: $\rho =0.784$, $\hbox {SE}=0.0054$). It is worth mentioning that while the Rasch and the 2HT model performed comparably under low and medium missingness, the 2HT performed worse than the Rasch model under high missingness. As above, all models performed worst when response bias was balanced (${\overline{\gamma }}=0.5$, GCMR: $\rho =0.874$, $\hbox {SE}=0.0070$, Rasch: $\rho =0.836$, $\hbox {SE}=0.0086$, 2HT: $\rho =0.827$, $\hbox {SE}=0.0095$) and best when bias was more pronounced (${\overline{\gamma }}=0.9$, GCMR: $\rho =0.904$, $\hbox {SE}=0.0053$, Rasch: $\rho =0.886$, $\hbox {SE}=0.0065$, 2HT: $\rho =0.886$, $\hbox {SE}=0.0062$). When missingness was high, the 2HT and Rasch model were found to be more affected by a decrease in bias than the GCMR ($\gamma = 0.9$ to 0.5, GCMR: $\Delta \rho =-0.084$, Rasch: $\Delta \rho = -0.102$, 2HT: $\Delta \rho = -0.105$), whereas SE increased similarly for all three models (GCMR: $\Delta$ $\hbox {SE}= .0144$, Rasch: $\Delta$ $\hbox {SE}=0.0130$, 2HT: $\Delta$ $\hbox {SE}=0.0135$).

### Real data study I: object-scene memory task in a web-based lifespan sample

#### Model comparison: LOO-CV

**Figure 2:**
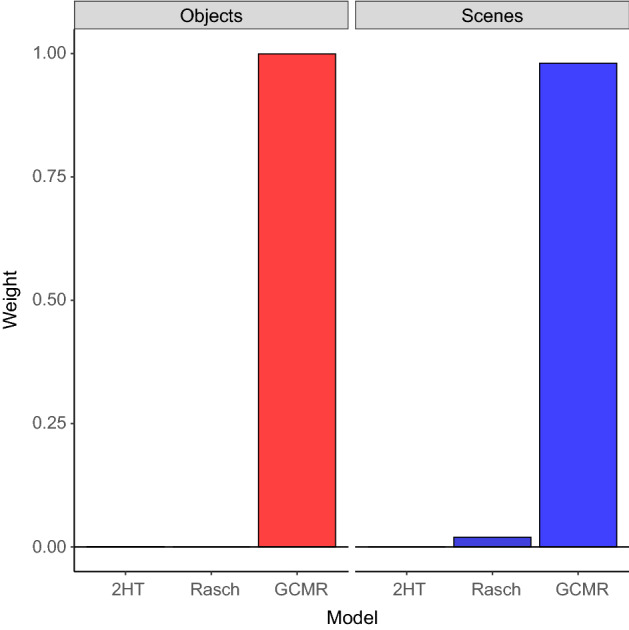
Leave-one-out cross-validation (LOO-CV) model weights obtained from Bayesian stacking. For both domains, $GCMR$ has highest stacking weight, suggesting best predictive performance for trial-based data in the object-scene task.

All models seemed to converge well (Fig. 8, Appendix C) as indicated by low ${\hat{R}}$ statistics^31^. As for the LOO-crossvalidation (Fig. 2), Bayesian stacking resulted in the GCMR obtaining almost all the mixing weight, both in the object condition ($w_{GCMR}=0.999$, $w_{2HT}=0.000$, $w_{Rasch}=0.001$), as well as the scene condition ($w_{GCMR}=0.980$, $w_{2HT}=0.020$, $w_{Rasch}=0.000$). This suggests that the GCMR has higher (out-of-sample) predictive power and model evidence than both Rasch and 2HT for trial-level data from this object-scene memory task.

#### Population analysis: linear mixed-effects model of point estimates

**Figure 3:**
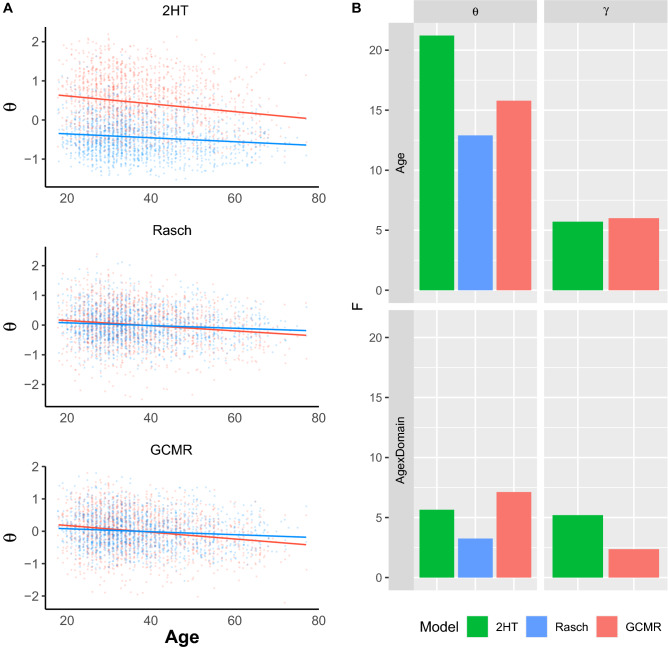
GCMR, Rasch and 2HT ability estimates across age. (**A**) The plot shows the age $\times$ domain interaction effect slopes for ability $\theta$ in all three models. While the main effect of age was observed in all models, only GCMR and the 2HT model showed an age $\times$ domain interaction. (**B**) $\theta$: While the 2HT shows strongest main effect of age, the GCMR shows strongest age $\times$ domain interaction effect. $\gamma$: Both models show general bias increase with age, while only the 2HT shows an interaction with domain.

In this study, we observed a negative main effect of age on ability $\theta$ for all three models (GCMR: $\textit{F} = 12.90$, $\hbox {p} = 0.0003$, Rasch: $\textit{F} = 15.79$, $\hbox {p} = 0.0001$, 2HT: $\textit{F} = 21.22$, $\hbox {p} < 0.0001$), as well as a robust age by domain interaction for both the GCMR and 2HT model (GCMR: $\textit{F} = 7.13$, $\hbox {p} = 0.0077$, 2HT: $\textit{F} = 5.65$, $\hbox {p} = 0.0176$). Only for the GCMR does the interaction survive Bonferroni correction, however. In the Rasch model, on the other hand, the interaction effect was not as robust (Rasch: $\textit{F} = 3.25$, $\hbox {p} = 0.0716$). The interaction effect was driven by steeper decline in object performance across age (Fig. 3A), which replicates our previous work suggesting that mnemonic discrimination of highly similar stimuli decreases with age, with an additional domain-dependency^1^. For an overview of the effects, see table 1. For the effects on bias parameter $\gamma$, see Supplementary information.

**Table 1:** Anova table $\theta$.

GCMR	Sum Sq	Mean Sq	NumDF	DenDF	F value	Pr (>F)
Age	7.79	7.79	1.00	1552.00	15.79	0.0001
Age:Domain	3.52	3.52	1.00	1552.00	7.13	0.0077
**Rasch**						
Age	7.09	7.09	1.00	1552.00	12.90	0.0003
Age:Domain	1.79	1.79	1.00	1552.00	3.25	0.0716
**2HT**						
Age	10.01	10.01	1.00	1552.00	21.22	0.0000
Age:Domain	2.66	2.66	1.00	1552.00	5.65	0.0176

#### Population analysis: latent regression

**Figure 4:**
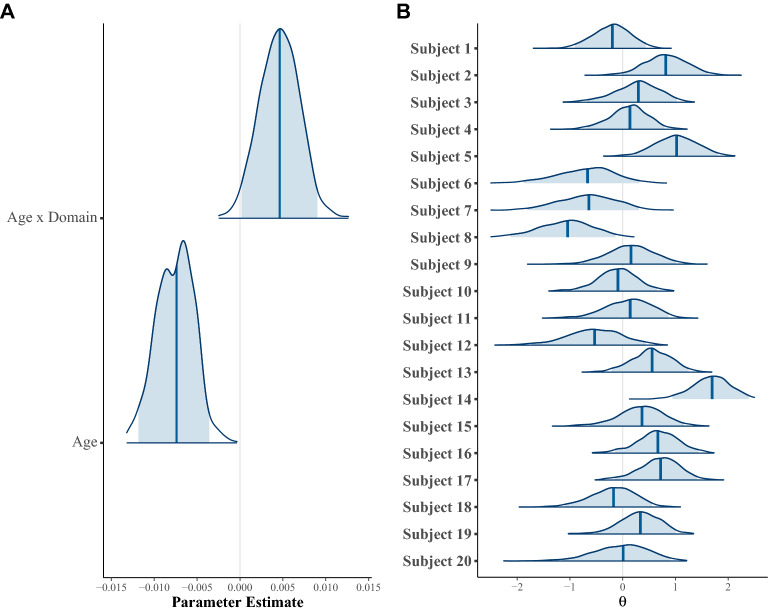
Posterior distribution of single subject ability (**A**) and group parameter estimates (**B**) from the latent regression extension of GCMR. (**A**) Posterior distributions of the coefficients for age and the age by domain interaction. For both parameters, the posterior distributions differ largely from zero (0 lies outside the credible interval). As with the LME analysis, we observe a negative effect of age ($\zeta$), and an age $\times$ domain ($\xi$) interaction. A positive interaction effect means that age-related decrease is stronger for objects. (**B**) Posterior distributions of ability estimates ($\theta$) for 20 randomly sampled subjects. Figure shows median (dark blue) and 95% credible interval (light blue).

Using this hierarchical model specification of the GCMR, we obtained the same pattern of results as with two-way procedure used above. In particular, we observe that posterior distributions for both the age and the age $\times$ domain estimates are mostly different from 0, in particular the credible intervals (using probability 0.95) do not overlap with 0 (see Fig. 4A), suggesting there is an effect of both age and age $\times$ domain. Unsurprisingly, the direction of the effects remains the same: a negative effect of age, and a stronger age-related decrease for object performance. Notice that we also obtain posterior distributions for each subject estimate, which reflect inter-individual differences in ability, Fig. 4B).

### Real data study II: CAT

**Figure 5:**
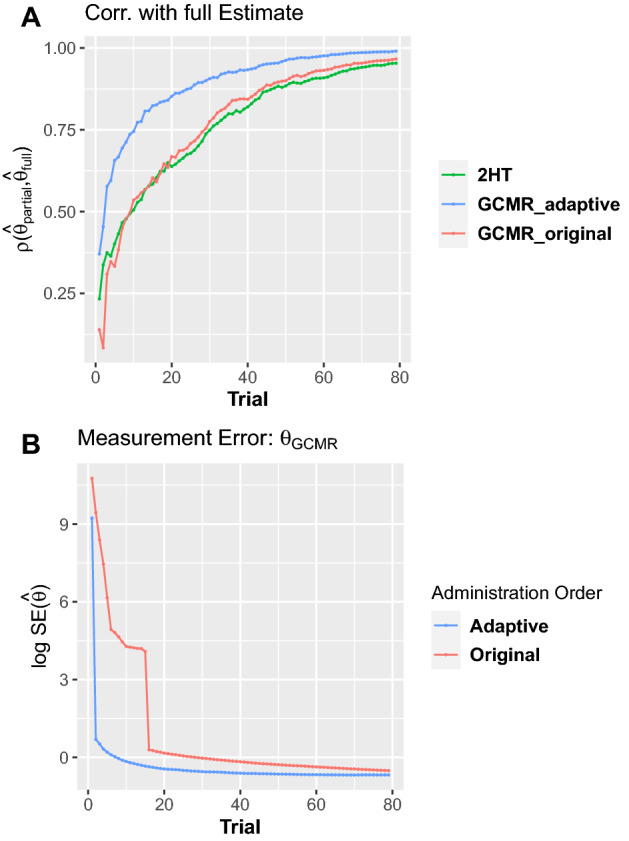
Trial-wise convergence of $\theta$ using original versus simulated adaptive item administration order. (**A**) While convergence was observed to be similar for GCMR and the 2HT model in the original test administration order, convergence of abilities $\theta _{GCMR}$ was faster under an adaptive order. (**B**) Standard error $SE_{\theta }$ decreases more rapidly in the adaptive than the original order, while both approaches converge in later trials.

When presenting stimuli in the original administration order, trial-wise correlation of parameter estimates with full-sample estimates increased faster for the GCMR than the 2HT model, reaching $\rho$=0.9 after 50 trials for $\theta _{GCMR}$ and 56 trials for $\theta _{2HT}$ (Fig. 5A). However, when an adaptive administration order was used, only 26 trials where necessary for $\theta _{GCMR}$ to reach $\rho =0.9$.

In order to compare trial-wise standard error of measurement ($SE_{\theta }$) for $\theta _{GCMR}$ in the original versus adaptive administration order (Fig. 5B), we measured how many trials were required to obtain a high confidence of ability estimates with $SE_{\theta }$ smaller than 0.6. While 79 trials were necessary to reach this criterion when using a non-adaptive order, only 26 trials were necessary using CAT.

## Discussion

In this paper, we introduce and validate a new General Condorcet Model for Recognition (GCMR) for dichotomous responses that combines aspects of the 2-High-Threshold (2HT) for recognition memory and the Rasch model from Item Response Theory^3,32^. Formally, the proposed GCMR model belongs to a more general class of General Condorcet Models^30^, with the restriction that the expected response is fixed by design rather than having to be estimated. The approach aims at overcoming the limitation of conventional recognition memory models that posit item homogeneity (e.g. 2HT, *d*$'$) by allowing for item difficulty to vary freely. GCMR is an IRT model, which separately parametrizes item difficulty and discrimination ability to enable ability estimation to be independent of the item set presented. Using both simulated and real data, we show that the proposed GCMR model outperforms both the Rasch an 2HT models in modeling old-new recognition memory data. We found favourable parameter recovery for the GCMR model and highlight cases in which ability estimates may be biased for both the Rasch and the 2HT model. Using real behavioural data from a mnemonic object-scene discrimination task, the GCMR also exhibited greater model evidence (using leave-one-out crossvalidation) compared to both the Rasch and 2HT model. Furthermore, face validity of GCMR was established by replicating previous findings of performance decline with higher age and a steeper decline in object compared to scene performance^1^. Moreover, we showed that group-level effects could be obtained within a hierarchical latent-regression model specification, without having to resort to a two-stage procedure. Finally, we demonstrated GCMR’s potential for adaptive testing, which demonstrates that using GCMR we can overcome severe limitations from conventional cognitive non-IRT models.

The approach contributes to recent work combining psychometric with cognitive models from recognition memory^24,25^, thereby drawing from advanced approaches to examine measurement precision as well as psychologically valid modeling of relevant latent variables. While Thomas^24^ and De Carlo^25^ both used SDT models as the basis of their cognitive models, to our knowledge GCMR is the first approach combining IRT with a recognition memory model from the MPT family aimed at assessing recognition memory. 2HT, as well as our proposed approach are particular instances of Binary MPTs (BMPT) called “Multitree MPTs”^27^, which have the property to involve different within-subject trial types (e.g. targets versus foils). Many formal properties of these models as well as non-Bayesian estimation methods using the Expectation-Maximization (EM) algorithm have been previously discussed^33^. GCMR might also be viewed as an example of what Batchelder^27^ referred to as “Rasch-BMPTs”, in which the transition probabilities between branches are given by a Rasch model. While Karabatsos and Batchelder^26^ applied this approach to a particular MPT called the general Condorcet model, our GCMR model is a more tailored adaptation for recognition memory modelling to overcome limitations of more conventional 2HT approaches. As such, GCMR differs from the two above SDT-based models in important ways. For instance, Thomas^24^ showed that a simple SDT model (*d*$'$) can be extended to a compensatory multidimensional 2P IRT model^34^. This model effectively places bias and memory discrimination under a common process, whereby one process can entirely compensate for another. It is expected that under (infinitely) high bias the discrimination may become almost unmeasurable, leading to response patterns that are solely predicted by the trial type (target versus foil). As an example, under such high bias the correct response probability for foils would approximate zero. The GCMR is non-compensatory in that way, since the discrimination and guessing are modeled as independent processes. That is both the guessing and perception process set a lower bound on performance. In the approach chosen by De Carlo^25^ and similar to the well-known 3PL IRT model, guessing or response bias is an item parameter while the GCMR defines it as a person-specific characteristic. We believe this to be a sensible and important feature of the present approach since guessing propensity has been shown to be influenced by clinical pathologies as well ageing, and might therefore serve as an important subject-specific behavioral marker in clinical assessment. However, GCMR represents but one effort to combine cognitive modeling with psychometric theory in order to assess recognition memory, and future empirical work is needed to determine which model is best suited to reflect the relevant latent processes.

The simulation study showed that parameter estimates of the 2HT and the Rasch model diverged strongly from GCMR ground truth under different manipulations of test difficulty, average response bias and data missingness, while the GCMR fared well in recovering ground truth parameters under these manipulations. One surprising observation was that a more biased guessing propensity did facilitate a more accurate estimation of ability, especially when the true ability was low in comparison to test difficulty. One might speculate that under low ability the amount of guessing increases, making ability estimation harder since more prone to noise. However, when guessing is highly biased, the variance it adds to responses is low, which again seems to facilitate ability estimation. Comparatively, the GCMR performed well under these less favorable conditions. This might be because it accounts for more sources of response variability than the two models separately, that is response bias, ability, and item difficulty simultaneously. This finding is especially relevant when studying clinical or populations in old age, as these often differ in terms of memory abilities^1,35–37^ and response bias^6^ compared to healthy young adults.

Using cross-validation our results demonstrate that the GMCR shows better out-of-sample prediction for trial-by-trial response data than the 2HT or the Rasch model. This suggests that since GCMR models item heterogeneity it may be suitable for behavioral modeling of trial-by-trial performance. Apart from adaptive assessment it may be used to model subjective trial difficulty and could be combined with measures from other modalities (e.g. fMRI). We replicated deteriorative effects of age on mnemonic discrimination ability previously reported by the authors^1^. One surprising finding is that the main effect of age was less significant for the GCMR than for the 2HT model. It may be that the effect is overestimated by the 2HT. Another possibility is that part of GCMR’s between-subject variance was modeled via the items. This might have arisen due to relatively weak item priors combined with few evaluations per item, and might have been biased by occasionally uneven item distributions across ability-related predictors such as age. One result showing the influence of free item parameters is that both the GCMR and Rasch model had weaker stimulus domain effects, being able to keep distributions for each domain around the prior mean, while the 2HT could not compensate for existing domain difficulty differences. This demonstrates the necessity to calibrate item parameters in a first step, when modeling subsamples independently. Unlike the 2HT model, GCMR is potentially able to model unbalanced item evaluations as long as their missingness is unrelated to ability, or predictors of ability (e.g. age). Even in such a case the GCMR can still be used if item parameters are calibrated on a suitable sample in advance. The domain-specific effect of age on ability was strongest for GCMR, which suggests that relevant variance regarding ability estimates was modeled. Domain-dependent age effects have been previously observed in other tasks assessing mnemonic discrimination^36,37^ and have been proposed to reflect distinct, domain-specific memory pathways^38^ and their age-related dysfunction^39,40^. As a follow-up to the above replication study, we explored a hierarchical, latent-regression extension of the GCMR, and found that group-level effects can be sensibly estimated in a single model (see Oravecz et al.^41^ for a related approach). In general, this approach allows to preserve and propagate uncertainty information in the person parameters across individual and group level, and should therefore provide more sensitivity than a two-stage analysis. The flexible MCMC estimation framework in Stan does also allow for further extensions to the model, such as adding more hierarchies by nesting participants within groups, or including further person- as well as item-regressors. In summary, we showed that GCMR may hold promise as a diagnostic tool in behavioral assessment of (domain-dependent) memory decline, and the current framework is able to incorporate both group- and subject-level estimates within a single model.

Finally, we demonstrated that GCMR might be useful for future studies to perform CAT in recognition memory tasks. However, unlike common IRT models focussing on a single person parameter we performed CAT on a model with two person parameters (ability and bias). To do so, a large set of items was calibrated and characterized using conventional item statistics from IRT. We were able to speed up convergence of ability estimation considerably by reducing the number of trials by 48% in order to reach 90% convergence. Real adaptive testing scenarios in future studies might prove to further speed up convergence. Here the algorithm could only choose items that were present in the actual data.

We also highlighted the benefits of obtaining trial-wise estimates of conditional measurement error and found that over trials the estimation error decreased significantly faster using an adaptive versus a non-adaptive item administration order. Based on these results CAT is promising in clinical research for its potential to make tests shorter and more efficient. As time-efficient test solutions are needed, this is particularly important for the growing field of mobile cognitive assessment. Regarding experimental work in recognition memory, the GCMR might contribute to the adaptive assessment of the well-studied cognitive variables memory ability and response bias in various populations.

It is important to mention some limitations and point out directions for future research. The complex structure of sparsity in our data set comes with costs when performing explorative analysis. Design sparsity prevented us from rigorous testing of further IRT assumptions such as conditional independence and dimensionality. It should be noted, however, that evidence in favour of two latent dimensions in recognition memory tasks has been found elsewhere^24^. Nevertheless, in future studies the GCMR might be tested on data from a more balanced design, where these assumptions can be easily checked. Moreover, the model in its current form assumes independence of ability and response bias, based on previous SDT studies showing that these variables can be manipulated separately^42^. However, those variables might be associated empirically. Given that ability and bias are currently modeled using independent distributions, it is difficult to directly model their relation. One solution might be to model both person parameters as jointly normally distributed, which would allow easy estimation of their covariance^41^. Finally, in its current form the model does not distinguish between various mnemonic sub-processes that have been proposed in the literature, such as item-familiarity^43–45^, context-familiarity^46,47^, or recollection^48,49^. In a similar vein, several recognition paradigms allow for more than the ’old/new’ responses, such as in the ‘Remember’/‘Know’/‘New’ procedure. Future modelling work should try and incorporate these aspects, which may also help obtain a more fine-grained assessment of potential underlying memory deficits.

In summary, we presented a GCM that accounts for varying item difficulty and is able to model trial-by-trial recognition memory performance better than the well-known 2HT model. Moreover, we were able to establish face validity by replicating earlier findings on recognition memory using the 2HT model. In addition, we showed that the results can be replicated using a single, latent-regression extension of the model, and briefly discussed the potential of such an approach. We then demonstrated that the GCMR can be readily applied in CAT, leading to refined measurement estimates and increased test efficiency. Lastly, we pointed out its great potential in the domain of clinical and mobile memory assessment.

## Methods

In what follows we briefly review the technicalities of the behavioral models from which we derive the GCMR and close this section with a presentation of our validation strategy using synthetic and real datasets.

### Behavioral models

We begin by describing the 2HT and the Rasch model more formally. The GCMR is then introduced, which integrates the two models and posits that the memory process is a function of both person ability and item difficulty, while also accounting for response biases.

#### 2HT model

In the context of recognition memory experiments, participants typically observe a series of (visual or auditory) stimuli. After each stimulus (or experimental trial), they are asked to indicate whether it was old or new. We further denote the participant’s response of a trial using random variable U taking value “old” or “new”. We further use variable *t* to indicate trial types taking on the values ${t} = target$ and ${t} = foil$. Then the 2HT (or “corrected-hits”) model specifies “hit” (correct recognition) and “false alarm” (false recognition) in terms of event probabilities as follows:

1 $$\begin{aligned}&P( hit ) = P( U = old | t = target ) = Pr + (1 - Pr)\gamma \end{aligned}$$

2 $$\begin{aligned}&P( \text{ false } \text{ alarm } ) = P( U = old | t = foil ) = (1 - Pr)\gamma , \end{aligned}$$

where *Pr* represents the discrimination parameter, and refers to the probability of “knowing” the correct answer, given that a target or foil was presented.

The guessing parameter $\gamma$ denotes the probability of responding “old” when the correct answer is unknown, reflecting a guessing tendency or response bias.

We can easily see that *Pr* is the difference of the hit rate and false alarm rate.

The proposed GCMR merges the 2HT and the Rasch model. As is common in IRT, the correctness of a response is modeled. For consistency, we reformulate the above 2HT model using variable $Y=1$ to code a correct response and $Y=0$ for an incorrect response

3 $$\begin{aligned} p(Y = 1|Pr,\gamma ,t) = Pr+(1-r) \gamma ^t (1-\gamma )^{(1-t)} \end{aligned}$$

in a trial with trial type $t=1$ for target trials and $t=0$ for foil trials. For the remainder, we will refer to *Pr* as $\theta _{2HT}$, where $\theta$ denotes an ability parameter.

#### Rasch model

Next we briefly describe the Rasch model^32^. Here, the probability of a correct response is assumed to be a function of both person ability and item difficulty. The model uses the logit function to map the real-valued ability and difficulty parameters to probabilities. The probability of answering correctly is given by the logit of their difference:

4 $$\begin{aligned} p(Y =1|\theta ,\beta )=\frac{e^{(\theta -\beta )}}{1+e^{(\theta -\beta )}}, \end{aligned}$$

where $\theta$ and $\beta$ refer to the person ability and item difficulty, respectively. The key assumption is that only the difference of both parameter affects probability of a correct response. The function approaches 1 when ability strongly exceeds item difficulty and 0 if the item is much too difficult for a certain person performing the task, e.g. on recognition memory. It is worth noting that neither trial type nor for response bias is accounted for.

#### GCMR—combining Rasch and 2HT model

The 2HT model assumes equal item difficulties, which is rarely the case in recognition memory tasks. The GCMR’s main purpose is to account for item heterogeneity. In order to do so, we can model the discrimination stage of the 2HT as depending on a latent person ability $\theta$ and an item difficulty $\beta$ given by the Rasch model (Eq. (4)), obtaining a model that accounts for person ability, item difficulty and response bias:

5 $$\begin{aligned} p(Y = 1|\theta ,\beta ,\gamma ,t)=\frac{e^{(\theta -\beta )}}{1+e^{(\theta -\beta )}}+(1-\frac{e^{(\theta -\beta )}}{1+e^{(\theta -\beta )}})\gamma ^t (1-\gamma )^{(1-t)} \end{aligned}$$

**Figure 6:**
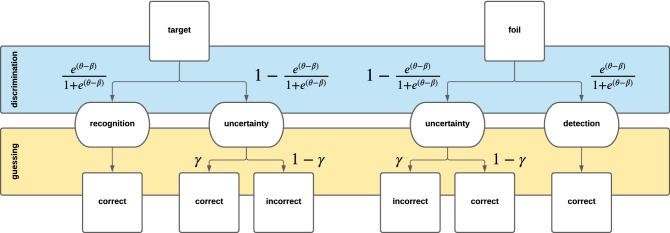
Schematic tree diagram of the GCMR. The proposed model can be interpreted using two decision trees, one for each trial type. The trees are divided into two latent processes: discrimination and guessing. Transition probabilities follow a Rasch model in the discrimination process, and are defined by parameter $\gamma$ in the guessing process.

The depicted model (Fig. 6) is equivalent to the Rasch extension of the GCM, with one important difference: unlike for the GCM, trial type *t* is not a parameter, but an experimental design variable. In other words, the expected response for each trial is set by the experimenter instead of having to be estimated. This is a common feature of recognition memory tasks, whereas GCM was conceived to infer the expected response as a function of cultural consensus in the domain of sociology and cross-cultural studies^30,41^.

### Model inference

Here we follow the Bayesian paradigm for estimation and model inference^50^, given that it has been successfully applied to models from the GCM family^26^. In addition, a Bayesian treatment allows for flexible incorporation of prior knowledge from previous studies recognition memory, which may be available once the model has been tested in different domains or samples. Moreover, Bayesian modeling provides an elegant way to analyse both parameter and model uncertainty, a very attractive feature in the context of clinical assessment. Here, the parameters for all models were estimated using Markov-Chain Monte-Carlo (MCMC) sampling, implemented in Stan via the R interface^51^. The use of MCMC sampling methods is omnipresent in the estimation of Bayesian models, as the integrals from the marginal distribution are typically intractable and cannot be estimated using conventional analytical methods^26^.

### Simulation study

We performed two validation studies to test face validity of the GCMR in the context of synthetic data, real data as well as its potential for adaptive testing. The goal of the simulation study was to assess the GCMR in scenarios of potential real studies, varying key parameters. We investigated under what conditions its ability estimates would align or differ from both the Rasch and 2HT model. Here, we focused on a descriptive presentation of the parameters used as well as the pattern of changes in parameter estimates under specific manipulations. As per construction our prior expectation was that the GCMR might capture information that the two single models do not provide. Furthermore, the manipulations tested here might help inform experimenters whether the use of GCMR is warranted for their specific data and problem at hand.

Concretely, we simulated response data of 100 subjects performing a task with 50 items (25 foils, 25 targets). A total of 20 simulations were run for each of the parameter combinations. We then estimated how well 2HT, Rasch and GCMR were able to recapture ground truth individual differences under different manipulations, including high bias, difficulty and missingness. For each model, we compared GCMR ground truth with estimated parameters using the Pearson correlation coefficient rho, since the model parameters live on different scales and hence a scale-free metric was warranted.

#### Default parameter values

Ability $\theta$ was modeled with a standard normal distribution, as is common in IRT, that is $\theta \sim N(0,1)$.

The bias parameter $\gamma$ in the GCMR is introduced via the 2HT model and the GCM, and there it is usually modeled as a probability on the unit scale, using the beta distribution as a prime candidate^26,41^. Here, we follow this parametrization. Given that the beta distribution has no explicit mean parameter, its mean $\mu _{\gamma }$ was varied indirectly by manipulating the distribution’s shape parameters $\alpha _{\gamma }$ and $\beta _{\gamma }$. In so doing, we varied its mean while keeping the variance constant. The three levels of bias mean $\mu _{\gamma }$ were modeled as follows:

**Table:** 

$$\mu _{\gamma }$$	
0.5	*Beta*(10, 10)
0.75	*Beta*(11.0625, 3.6875)
0.9	*Beta*(5.904, 0.656)

#### Varying test difficulty and response bias

The first manipulation aimed to investigate the potential key advantage of GCMR compared to the Rasch and 2HT model, namely accounting for item difficulty in addition to trial type and response bias. We were interested in how parameter estimation is affected when overall performance approaches chance level. Low performance is likely to be encountered in clinical populations as well as ageing cohorts, when these are compared to healthy young subjects on the same test. Furthermore, diverging patterns of response bias have been observed in these cohorts. Specific failure to correctly estimate ability under low performance or diverging response bias is therefore of paramount importance to clinicians. Here we varied average test difficulty as a means to manipulate performance, and explored how the effect of difficulty would interact with varying levels of response bias.

The true parameter values of average item difficulty $\mu _{\beta }$ and average response bias $\mu _{\gamma }$ were varied as follows:

**Table:** 

$\mu _{\beta }$	$\mu _{\gamma }$
$-2$	0.5
0	0.75
2	0.9

Parameter levels were crossed, leading to a grid of 9 parameter combinations.

#### Varying missingness and response bias

One fundamental limitation of the 2HT model is that person ability and item- or test difficulty are not necessarily independent. Thus $\theta _{2HT}$ does not define a test-independent latent construct (dimension) as is aimed for in IRT^52^. As a consequence, 2HT model estimates are only comparable when using the same or a truly parallel test. Having to compare participants performance across different tests is a situation well familiar to many experimenters. This can arise when participants do not respond to all items, or in the context of longitudinal assessment. In this part of the simulation study we manipulated the amount of missing data, effectively creating varying subtests.

Here, we tested a simple kind of missingness, where a fixed number of trials per participant were randomly deleted. Note that this should not change the mean test difficulty, but its variance. We expected that this would create more noise in $\theta _{2HT}$ estimates given its dependence on test difficult, as compared to ability estimates from the GCMR and possibly the Rasch model, which both directly account for item difficulty. To test this, the percent of missing data per subject p(missing) and the true parameter values of $\mu _{\gamma }$ were varied as follows:

**Table:** 

p(missing)	$\mu _{\gamma }$
0	0.5
0.25	0.75
0.5	0.9

For model estimation, we used the priors as specified in Appendix A.

### Real data study I: object-scene memory task in a web-based lifespan sample

In order to validate the GCMR on real data, we applied it to a large sample dataset^1^ in which participants performed a recognition memory task^35^. In this particular study, participants also performed the task on differing subsets of a large item pool. The use of (pseudo-)random subsets of a larger stimulus pool is a common strategy in recognition memory research to minimize learning and stimulus-repetition effects^53,54^. Thus, this study represents a common application scenario characterized by high missingness (or sparsity). As mentioned earlier, for conventional estimates such as $\theta _{2HT}$ to be meaningful, equal test difficulty must be assured. The data set used here very likely violates the assumption of equal difficulty of all the subtests. GCMR on the other hand does not rely on tests being equally difficult, as person and item parameters are estimated independently. Moreover, by modeling item heterogeneity, the GCMR is able to capture trial-by-trial variance, which cannot be predicted by the 2HT model.

Here, we first compared model evidence using Bayesian model stacking, predicting that GCMR would show better (out-of-sample) predictive performance than both the 2HT and Rasch model. We then explored face validity of GCMR, investigating whether the pattern of results previously found with conventional 2HT modeling^1^. In particular, the study found that task performance decreases with age, and that the age-related decrease depends on the stimulus domain, being stronger for stimuli representing objects than for scene stimuli.

#### Sample and stimulus material

We used a large web-based observational study of 1554 adults (18-77 years, M = 37.19, SD = 11.61, 61% females) that was analyzed by our group previously^1^. The study had been approved by the ethics committee of the Otto-von-Guericke University, Magdeburg. All subjects had given informed consent for their participation by explicitly ticking an “I agree” box on the consent form, following ethics and data security guidelines of the Otto-von-Guericke University. Participants had on average only responded to 2.42% of stimuli of a large item-pool, resulting in a highly sparse response matrix (indicating high missingness). They were tested on previously fixed subsets of the entire item pool, which themselves were subject to further randomizations of the stimulus versions presented. Therefore, missingness was induced by design, given that the following quantities varied across participants: (1) the item overlap between subsets, (2) the number of items tested. Consequently, this resulted in a rather unbalanced study design. Consequently, a larger sample increases numbers of available responses for each stimulus to eventually result in more reliable item parameters.

#### Task

Participants performed a 2-back mnemonic discrimination task, in which they had to discriminate between target and lure versions of object and scene stimuli. It constitutes a version of the object-scene task that we had previously employed^35^.The task consisted of several trials containing two item presentations followed by two identical (target) or very similar different (lure) test items each. Subjects had to respond to each test stimulus with old/new judgments pressing the left or right arrow key, whereby each trial either contained only objects or scenes.

#### Model comparison

To assess model fit, we used efficient approximate leave-one-out (LOO) cross-validation for Bayesian models using Pareto smoothed importance sampling (PSIS)^55^. The aim of cross-validation is to test out-of-sample prediction and penalize model complexity. We compared the three models (GCMR, Rasch, 2HT) using Bayesian stacking, which maximizes the leave-one-out predictive density of the combination distribution, finding the optimal linear combining weights that maximize the leave-one-out log score^56^.

This model comparison approach identifies the best model as the one obtaining the highest stacking weight.

#### Population analysis: linear mixed-effects model of point estimates

Posterior means of ability parameters from the GCMR, Rasch and 2HT model were separately estimated for task data from each domain (objects and scenes). Estimates were then pooled, scaled and centered for each model and subsequently entered into a group analysis. Here we used linear mixed effects modeling with the lme4 package in R^57^, in order to assess effects of age and the age $\times$ stimulus domain interaction. We also included stimulus domain as a covariate of no interest. Random intercepts were included for each participant. This enables accounting for the nested nature of the study design, including repeated measures for each subject. For hypothesis testing, we corrected for multiple comparisons (3 × age+3 × age $\times$ domain), obtaining a bonferroni-corrected $\alpha$-level of $0.05/6 = 0.0083$.

#### Population analysis: latent regression

In addition to the common two-stage procedure above, where point estimates are extracted from the first-level analysis for subsequent regression with covariates of interest, we show that the GCMR can be extended hierarchically to accommodate further covariates. In the present example, this allowed us to directly model the effect of age, stimulus domain, and their interaction. The full model can be written as

6 $$\begin{aligned} p(Y =1|\theta ,\beta ,\gamma ,\xi ,t)=\frac{e^{(\theta -\beta +\xi \times age\times domain)}}{1+e^{(\theta -\beta +\xi \times age\times domain)}} +\left( 1-\frac{e^{(\theta -\beta +\xi \times age\times domain)}}{1+e^{(\theta -\beta +\xi \times age\times domain)}}\right) \gamma ^t (1-\gamma )^{(1-t)}, \end{aligned}$$

where $\xi$ denotes the age $\times$ domain interaction effect. Furthermore, we specified ability and item difficulty as hierarchically depending on age and domain, respectively. Specifically, the parameter priors were set as follows: $\theta \sim N(\zeta \times age,1)$ and $\beta \sim N(\delta \times domain,1)$. Here $\zeta$ denotes the age effect, and $\delta$ is the effect of stimulus domain.

The covariate “age” was centered, and the “domain” covariate was coded as $-0.5$ for objects and 0.5 for scenes. As for the parametrization, the priors’ standard deviations were adjusted for the covariates’ scale using the formula 2.5/*sd*(*covariate*); see Appendix A for a detailed prior specification.

### Real data study II: CAT

In this last experiment, we explored the potential of the GCMR to be used in CAT. CAT requires item difficulty to be modeled and adaptively matched with a participant’s ability in a trial-by-trial fashion. This requires modeling of item parameters and cannot be performed using conventional detection models such as the 2HT model. Notice that unlike the Rasch model, GCMR possesses two person-specific parameters (i.e. ability and bias) which are fitted simultaneously, and can therefore be seen as a multidimensional IRT model. Here, we tested in a second application study whether (A) this parameter inference can be reliably performed; and whether (B) CAT can accelerate parameter convergence compared to conventional fixed tests. For one thing, this may significantly improve test efficiency for clinical applications.

#### Sample

We focused on the same web-based sample as in the above real data study. In a first step, 200 participants (denoted as the CAT sample) were sampled at random for inclusion in the adaptive test experiment. Data from the remaining 1354 participants (calibration sample) was used to calibrate the item sets.

#### Item calibration

**Figure 7:**
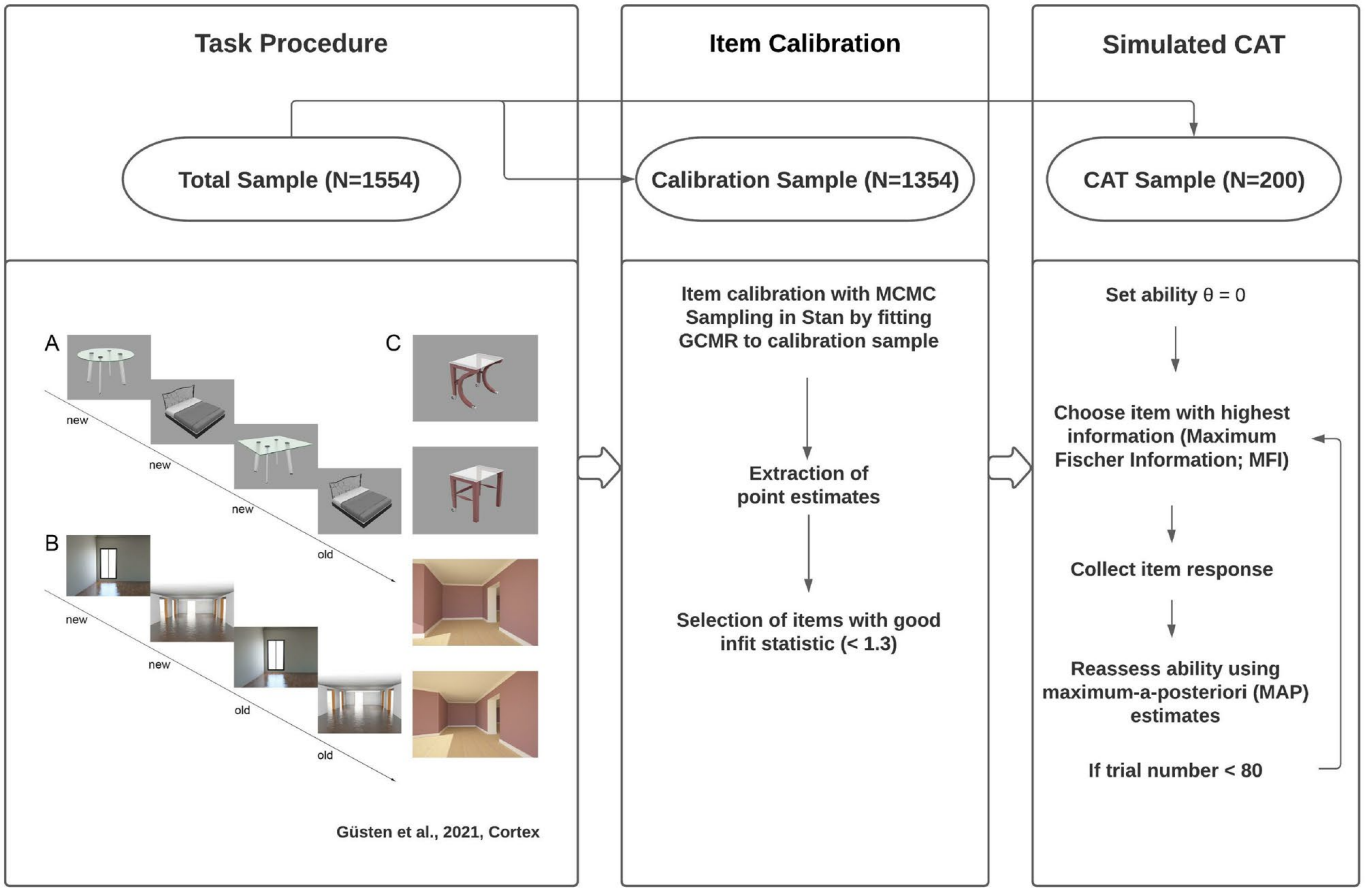
Schematic overview of Simulated CAT Procedure. *Task Procedure* Task data was taken from Güsten et al., 2021. Participants were presented pictures of objects (**A**) or scenes (**B**), in trials of 4 stimuli. Each trial consisted of two new item presentations (presentation phase) followed by two further presentations (test phase), in which stimuli were either exact repetitions or similar lures (**C**). In the test phase, participants had to respond “old” to repetitions and “new” to lures. *Item Calibration* Data from the calibration sample was fit to the GCMR model, item estimates were extracted and suitable items for CAT were selected. *Simulated CAT* Short summary of the simulated CAT algorithm.

**Figure 8:**
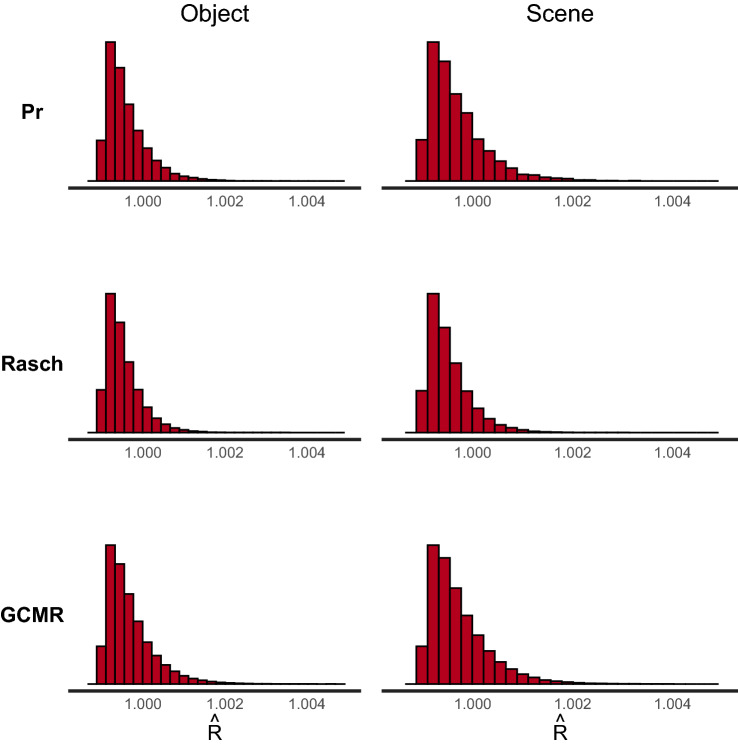
Model convergence. All three models converged well for both domains. An $\hat{R}$-statistic of well below 1.1 indicates that parameter estimates were consistent across MCMC chains.

As commonly done in CAT, we restricted the test to a subset of items that showed good model-data fit (see Fig. 7 for a schema of the CAT procedure) . Item parameters were fitted using the calibration sample and estimated via MCMC sampling in Stan, and posterior mean values subsequently extracted as point estimates of the parameters. Given that a sufficient amount of trial data per participants was needed for the CAT algorithm, a single model was fitted on the combined object and scene data. For item selection, we used the common infit statistic^58^, for which scores around 1 or lower indicate a good model-data fit. Underfitting items were excluded based on an infit threshold of 1.3^58^.

#### CAT procedure and algorithm

Response data from each subject of the CAT sample was used to simulate an adaptive test, mainly following the strategy by Reise and Henson^22^. All participants were given a starting latent ability of $\theta = 0$. Then, the item with the highest information given that trait level was administered (see item information function in Appendix B). The algorithm, which was based on the CatR toolbox^59^ and modified to incorporate the GCMR, used Maximum Fischer Information (MFI) as the item choice criterion. Given the participant’s response, a new ability was assessed using maximum-a-posteriori (MAP) estimates^60^, with ability priors being standard normally distributed. The above procedure of computing the item information at the participant’s current estimated ability, presenting the most informative item, collecting the response and reestimating ability was repeated for 80 trials. The CAT was restricted to this length so as to remain comparable to common paradigms in the field. Also, further extending the length would have prevented the algorithm to choose adaptively fitting items, given that item presentations per participants were set by the existing data (M=130.67, SD=3.23).

#### Convergence analysis

In order to evaluate the efficiency of the approach, we calculated the following two statistics after every CAT trial^22^: (1) the correlation of individual trial-wise updated parameter estimates with the corresponding full-sample estimates and (2) trial-wise standard errors of ability estimates ($\theta$). Notice that CAT estimates were always based on up to 80 trials, while the full estimates were based on the entirety of trials seen by the participant in the original data.

As noted earlier, CAT cannot be performed with conventional recognition models such as the 2HT. In order to show the benefit of using CAT, we calculated trial-wise ability estimates of GCMR and 2HT using the original, non-adaptive item administration order for comparison. We then assessed to what extent the increase of trial-wise GCMR ability correlation with full estimates can be accelerated by an adaptive administration order.

### Supplementary Information

Supplementary Information.

## Data Availability

The Stan model code, as well as the scripts for the simulation and population studies are available at https://github.con/jguesten/GCMR. Anonymized data from the population study will be shared upon request for the sole purpose of replicating the current findings.

## Appendix A

### Prior specification

**Rasch**

$$\begin{aligned}&\theta \sim N(0,1)\\&\beta \sim \mu _{\beta }+\sigma _{\beta }\cdot \alpha _{\beta }\\&\mu _{\beta } \sim cauchy(0,5)\\&\sigma _{\beta } \sim cauchy(0,5)\\&\alpha _{\beta } \sim N(0,1) \end{aligned}$$

**2HT**

$$\begin{aligned}&\theta \sim N(0,1)\\&\gamma \sim Beta(\alpha _{\gamma },\beta _{\gamma })\\&\alpha _{\gamma } \ge 0\\&\beta _{\gamma } \ge 0 \end{aligned}$$

**GCMR**

$$\begin{aligned}&\theta \sim N(0,1)\\&\beta \sim \mu _{\beta }+\sigma _{\beta }\cdot \alpha _{\beta }\\&\gamma \sim Beta(\alpha _{\gamma },\beta _{\gamma })\\&\mu _{\beta } \sim cauchy(0,5)\\&\sigma _{\beta } \sim cauchy(0,5)\\&\alpha _{\beta } \sim N(0,1)\\&\alpha _{\gamma } \ge 0\\&\beta _{\gamma } \ge 0 \end{aligned}$$

**Latent-regression GCMR**

$$\begin{aligned}&\theta \sim N(\zeta \times age,1)\\&\beta \sim \mu _{\beta }+\sigma _{\beta }\cdot \alpha _{\beta }\\&\gamma \sim Beta(\alpha _{\gamma },\beta _{\gamma })\\&\mu _{\beta } \sim cauchy(0,5)\\&\sigma _{\beta } \sim cauchy(0,5)\\&\alpha _{\beta } \sim N(\delta \times domain,1)\\&\alpha _{\gamma } \ge 0\\&\beta _{\gamma } \ge 0\\&\zeta \sim N(0,0.215)\\&\delta \sim N(0,0.504)\\&\xi \sim N(0,0.43)\\ \end{aligned}$$

Notice that we used a hierarchical prior for beta. Furtermore, the parameterization was non-centered, in order to facilitate convergence of Hamiltonian Monte Carlo Sampling in Stan^61^. This is possible for Gaussian distributions, since

$$\begin{aligned} \alpha \sim N(0, 1) \implies \mu + \sigma \cdot \alpha \sim N(\mu , \sigma ) \end{aligned}$$

## Appendix B

### Item information function with respect to ability $\theta$

$$\begin{aligned} I_{\theta }(\theta ,\gamma ) = \frac{[\frac{\partial }{\partial \theta }P(\theta ,\gamma )]^2}{P(\theta ,\gamma )\cdot (1-P(\theta ,\gamma ))} \end{aligned}$$

$P(\theta ,\gamma )$ refers to the probability of a correct response provided in Eq. (5).

## Appendix C

See Fig. 8.
