# Supplementary material for: Bayesian modeling of item heterogeneity in dichotomous recognition memory data and prospects for computerized adaptive testing
Source: Sci Rep. 2022 Jan 24;12:1250. doi: 10.1038/s41598-022-04997-3 (PMC8786965; doi:10.1038/s41598-022-04997-3)
Supplement: Supplementary file 1 — Supplementary Information. [file 41598_2022_4997_MOESM1_ESM.pdf]

Supplementary information

Anova Table γ

GCMR	Sum Sq	Mean Sq	NumDF	DenDF	F value	Pr (>F)
Age	1.89	1.89	1.00	1552.00	6.00	0.0144
Age:Domain	0.75	0.75	1.00	1552.00	2.36	0.1244
2HT						
Age	1.92	1.92	1.00	1552.00	5.71	0.0170
Age:Domain	1.75	1.75	1.00	1552.00	5.20	0.0228
